# What works in falls prevention in Asia: a systematic review and meta-analysis of randomized controlled trials

**DOI:** 10.1186/s12877-017-0683-1

**Published:** 2018-01-05

**Authors:** Keith D. Hill, Plaiwan Suttanon, Sang-I Lin, William W.N. Tsang, Asmidawati Ashari, Tengku Aizan Abd Hamid, Kaela Farrier, Elissa Burton

**Affiliations:** 1 School of Physiotherapy and Exercise Science, Curtin University, GPO Box U1987, Perth, WA 6845 Australia; 2 Physical Therapy Department, Faculty of Allied Health Sciences, Thammasat University, Bangkok, Pathumthani Thailand; 3 Department of Physical Therapy, National Cheng Kung University, Tainan, Taiwan; 4 Department of Rehabilitation Sciences, Hong Kong Polytechnic University, Hung Hom, Hong Kong; 5 Malaysian Research Institute on Ageing, Universiti Putra Malaysia, Serdang, Selangor Darul Ehsan Malaysia

**Keywords:** Falls prevention, Effectiveness, Asia, Elderly, Community

## Abstract

**Background:**

There is strong research evidence for falls prevention among older people in the community setting, although most is from Western countries. Differences between countries (eg sunlight exposure, diet, environment, exercise preferences) may influence the success of implementing falls prevention approaches in Asian countries that have been shown to be effective elsewhere in the world. The aim of this review is to evaluate the scope and effectiveness of falls prevention randomized controlled trials (RCTs) from the Asian region.

**Method:**

RCTs investigating falls prevention interventions conducted in Asian countries from (i) the most recent (2012) Cochrane community setting falls prevention review, and (ii) subsequent published RCTs meeting the same criteria were identified, classified and grouped according to the ProFANE intervention classification. Characteristics of included trials were extracted from both the Cochrane review and original publications. Where ≥2 studies investigated an intervention type in the Asian region, a meta-analysis was performed.

**Results:**

Fifteen of 159 RCTs in the Cochrane review were conducted in the Asian region (9%), and a further 11 recent RCTs conducted in Asia were identified (total 26 Asian studies: median 160 participants, mean age:75.1, female:71.9%). Exercise (15 RCTs) and home assessment/modification (*n* = 2) were the only single interventions with ≥2 RCTs. Intervention types with ≥1 effective RCT in reducing fall outcomes were exercise (6 effective), home modification (1 effective), and medication (vitamin D) (1 effective). One multiple and one multifactorial intervention also had positive falls outcomes. Meta-analysis of exercise interventions identified significant benefit (number of fallers: Odds Ratio 0.43 [0.34,0.53]; number of falls: 0.35 [0.21,0.57]; and number of fallers injured: 0.50 [0.35,0.71]); but multifactorial interventions did not reach significance (number of fallers OR = 0.57 [0.23,1.44]).

**Conclusion:**

There is a small but growing research base of falls prevention RCTs from Asian countries, with exercise approaches being most researched and effective. For other interventions shown to be effective elsewhere, consideration of local issues is required to ensure that research and programs implemented in these countries are effective, and relevant to the local context, people, and health system. There is also a need for further high quality, appropriately powered falls prevention trials in Asian countries.

**Electronic supplementary material:**

The online version of this article (10.1186/s12877-017-0683-1) contains supplementary material, which is available to authorized users.

## Background

Falls are recognized as a major cause of death and growing burden of disease world-wide [1, 2]. There has been strong growth in the available research evidence from randomized controlled trials investigating interventions to reduce falls among older people living in the community, with the most recent (2012) Cochrane review reporting 159 studies with 79,193 participants [3]. However, data from several developed countries indicate that despite this high volume of quality research, that key national indicators such as rates of falls related hospitalizations continue to increase (for example, in Australia, trend data from 1999 to 2013 indicate an average 2% increase in age-standardized fall related hospitalizations per year) [4–6]. Importantly, if rates of fall related hospitalizations remain stable or increase, then with aging populations this means substantial growth in actual case numbers of people being hospitalized due to falls (see Fig. 8.3 in [4]).

Population aging is occurring at differing rates between countries [7]. Japan is the “oldest” country in the world, with 26% of its population aged over 65 years [8]. Many developed countries already have more than 15% of their population aged greater than 65 years, [8] and are expected to exceed 20% by 2050. In contrast, many developing countries in Asia such as Malaysia, Thailand, Indonesia, Cambodia, Vietnam and the Philippines have less than 10% of their population aged more than 65 years [9, 10]. However, Asia is home to 60% of the world’s population, [8] and has the fastest aging population of any region in the world [11]. There is a need for a strong preventive approach to minimize the risk of falls and associated injuries as countries’ aging populations grow.

The available research provides mixed evidence about the magnitude of the problem of falls between countries. While it is widely accepted from prospective, large scale representative samples in a number of Western countries that between 30 and 39% of the population aged greater than 65 will experience one or more falls in a 12 month period, [12–14] the proportion of older people reporting falls in the limited number of studies in Asian countries has generally been lower and considerably varied (14–34%, median 18%) [15–17]. Limitations to the design of most of these studies in Asian countries include utilizing retrospective data collection (recall of falls in the preceding 12 months), and lack of representative sampling. There may also be some cultural factors influencing whether or not a fall will be reported by an older person, [18] which may contribute to varying and under-reporting of falls outcomes.

Factors contributing to falls risk may also vary between countries and cultures [18, 19]. Differences in intrinsic factors may include sunlight exposure, diet, stature, exercise patterns and preferences, and knowledge and attitudes towards ageing and falls prevention. For example, in a recent qualitative study of older people in Thailand, falls prevention was not noted as a perceived benefit of exercise, and some family and cultural values were considered to be potential barriers to older people becoming involved in exercise [20]. Another qualitative study in India reported that older people often considered falls were random events, and not considered a health priority [21]. A study in China reported fatalistic perceptions about falls being common among older people, low levels of knowledge about falls prevention interventions were evident, and falls were often hidden from family and doctors, and were not often discussed openly [22]. Differences in extrinsic factors also exist, including the home and outdoor environment, and footwear [17, 21, 23]. Type of housing and flooring surfaces vary substantially across Asia, and outdoor environments such as footpaths are often poorly maintained or non-existent. A study in China highlighted environmental factors such as adequate lighting on stairs and adequate step width as protective of falls (and therefore poor lighting and narrow step width as risk factors for falls) [17]. Additionally, there can be differences in health services and systems, and engagement of older people in these. Focus is often on treatment of acute health conditions, and less on preventative care.

The growing evidence highlighting different rates of falls and potential falls risk factors between countries raises the question of whether interventions that have been shown to be effective in one country may need some tailoring if being introduced into countries that have considerable diversity to the country where the intervention was shown to be effective. One recent scoping review has considered this issue in the context of falls prevention studies that have been conducted in South East Asian countries [23]. This review reported some unique aspects of socioeconomic, geographical and cultural differences of South East Asian countries, and identified limited quality research investigating falls prevention interventions in this region. However, the majority of the studies reported were non-randomized trials, studies were limited to only South East Asian countries, and there was no quality assessment of the included studies. The objective of our systematic review paper was to review the *randomized controlled trial* research evidence conducted among older people living in the community across the Asian region to identify (1) the type and number of falls prevention interventions shown to be effective in Asian populations; and (2) gaps for future research investigating falls prevention interventions in Asian countries.

## Methods

The 2012 community setting falls prevention Cochrane review was used as the basis for this review as it is the most recent and extensive systematic review and meta-analysis available on this topic [3]. Studies in the Cochrane review were categorized as to whether the study was undertaken in an (i) Asian, or (ii) non-Asian country. Additionally, a systematic review of the falls prevention literature published since the 2012 Cochrane review was also conducted, to identify more recent falls prevention RCTs conducted in the Asian region. This update component was guided by the Additional file 1: PRISMA checklist to ensure the results are reported systematically [24].

Intervention types were classified according to the Prevention of Falls Network Europe (ProFaNE) classification as (i) single interventions, (ii) multiple interventions (two or more single interventions, applied to all participants), and (iii) multifactorial interventions (two or more interventions, targeted to an individual’s risk factor profile, often based on a falls risk assessment process – different participants receive a different mix of interventions) [25]. Single interventions were further classified as described in the ProFANE classification as (a) exercise, (b) medication, (c) psychological, (d) environmental/assistive technology, (e) interventions to increase participant knowledge, (f) surgical interventions (eg cataract surgery, cardiac pacemaker surgery), (g) interventions to address incontinence, and (h) fluid or nutritional therapy. This intervention classification system was used in the 2012 Cochrane review, [3] and was also used to classify the more recent RCTs.

### Eligibility criteria for RCTs published since 2012 Cochrane review

The additional studies included in this review met the following eligibility criteria: peer reviewed articles published from January 2012 – November 2016; studies undertaken in Asia; randomized controlled trials; people aged 60 years and over – at least 50% of the sample; living in the community, and reporting at least one falls outcome.

### Information sources for additional RCTs

Studies were identified from six databases: Medline (Proquest), CINAHL, PubMed, PsycINFO, SPORTDiscus and Scopus for the time period described above. Reference lists from the included articles were also scanned. Only papers in English were included, no unpublished data, conference proceedings, books, poster abstracts or theses were included.

### Search strategy for additional RCTs

The search strategy used a mix of keywords which could be identified in the title and/or abstract. The search strategy included fall* [Title/abstract] AND communit* [Title/abstract] AND RCT [Title/abstract] OR randomi* controlled trial [Title/abstract]. There were differences between the databases for language and syntax, and where this occurred only the abstract was searched.

### Study selection for additional RCTs

Study selection was a three-stage process, stage one involved one author (KF) screening all of the additional identified papers based on their titles; stage two involved screening abstracts according to the eligibility criteria; and stage three involved two independent researchers (KF and EB) screening full articles. Where disagreements occurred the two researchers discussed the reasons for their decision, referring back to the eligibility criteria throughout the process, until they reached consensus.

### Data collection process

Characteristics of the intervention programs undertaken in Asian countries were retrieved from original publications independently by two of the researchers (KH and EB/KF). Comparing characteristics of the interventions (eg for exercise – type, duration, frequency) provided an opportunity to explore factors that may have contributed to the success or failure of different interventions in Asian populations.

### Study quality assessment for additional RCTS (post 2012 Cochrane review)

The Cochrane Collaboration’s risk of bias tool [26] was used by two researchers (KF and EB) independently to assess methodological quality of each paper (study quality of the Cochrane review papers can be accessed in the Cochrane review). Categories assessed by the tool include sequence generation, allocation concealment, blinding (staff, participants and outcome assessors), incomplete outcome data, selective outcome reporting and other sources of bias [26]. Risk of bias was assessed as “low risk”, “medium risk” or “high risk” [26]. Where disagreement occurred between the two assessors a third assessor (EJB) also assessed those domains, discussed with the two independent assessors their outcomes and came to a consensus.

### Analysis

Extracted data from all the Asian studies were tabulated, and overall sample demographics and characteristics of the interventions undertaken in Asian countries summarized, to assist in interpretation of the primary outcomes.

The primary analyses for this paper involved meta-analysis of falls outcomes (e.g. number of falls) by intervention type (according to the ProFaNE classification) where there were two or more randomized controlled trials conducted in Asian countries with comparable data.

For the meta-analyses, number of participants and events (falls, fallers, non-injurious falls) for each group were sourced from all original articles (including Cochrane review articles). RevMan 5.3 software was used to conduct the analyses and generate the forest plots, using a Mantel-Haenszel’s fixed effect model (with odds ratios and 95% confidence intervals calculated) [27]. Visual inspection of the forest plots and the I^2^ statistic were used to assess heterogeneity. When heterogeneity was deemed as high (I^2^ > 50%) a random effects model was applied. Where a study had two intervention groups and one control group, the intervention groups (i.e. dichotomous data) were summed (as recommended by Cochrane) for both the outcome (e.g. number of falls) and the sample size independently [27]. Subgroup analysis was undertaken where two or more programs/services within the intervention type could be grouped, for example Tai Chi within exercise interventions. Studies with group differences in baseline characteristics were omitted. Statistical significance was considered at *p* ≤ 0.05. Some studies reported more than one single intervention arm compared to a control arm. In these cases, if separate falls data were reported for each of the single interventions, then these were reported separately in the tables and meta-analyses.

## Results

Of the 159 randomized controlled trials reported in the 2012 Cochrane review [3], 15 (9%) were conducted in Asian countries (Japan, *n* = 6; [28–33] Taiwan *n* = 6; [34–39] Thailand, *n* = 2; [40, 41] and China/Hong Kong, *n* = 1 [42]). The remaining 144 published randomized trials were from non-Asian countries, including the United States of America (*n* = 34, 21%), Australia (*n* = 27, 17%), the United Kingdom (*n* = 27, 17%), Canada (*n* = 12, 8%), the Netherlands (*n* = 9, 6%), New Zealand (*n* = 6, 4%), Germany (*n* = 6, 4%), and other parts of Europe (*n* = 16, 11%). Several other studies were conducted across two or more countries (*n* = 4), although none of these included countries in Asia. Table 1 reports the number of randomized trial interventions classified by ProFaNE classification type, between Asian and non-Asian populations.

**Table 1:** Study types based on the ProFaNE intervention classification, for randomized trials conducted in Asia, and elsewhere in the world

	Number of randomized controlled trials (RCTs) in the 2012 Cochrane review				Additional RCTs conducted in Asia post 2012 Cochrane review	RCTs conducted in Asia (in the Cochrane review, and published subsequently) with significant improved falls outcome^#^
INTERVENTION TYPE	Asia	Rest of world	Total	Cochrane review conclusion (one or both falls outcomes) – all countries (see Footer for notes re ✓ / x)	Asia	Asia
Single intervention						
Exercise (includes studies that had other types of interventions in separate arms of the study, relative to a control group, so long as results were reported separately for comparison of the exercise and control interventions)	8 [28, 29, 31–34, 38, 42]	51	59	• Multi component group exercise ✓ • Multi component home exercise ✓ • Tai Chi ✓	7 [43–47, 49, 52]	• Multi-component group exercise ✓ • Tai Chi ✓ • Multi-target stepping program/obstacle course program ✓
Medication (drug target)	1 [30]	15	16	• Vitamin D supplementation (for those with baseline low vitamin D) ≈ • Psychotropic withdrawal ✓ • Prescribing modification ✓	0	• Vitamin K2, vitamin D2 and calcium supplementation (combined) ✓
Surgery (including cataract surgery, cardiac pacing)	0	5	5	• Cataract surgery (first eye) ✓ • Cardiac pacing (for those with carotid sinus hyper-sensitivity) ✓	0	x
Fluid or nutrition therapy	0	3	3	x	0	x
Psychological interventions (including cognitive behavioral therapy)	1 [37]	1	2	x	0	x
Environment / assistive technology	1[38]	12	13	• Home safety assessment and modification ✓ (more effective for those with high falls risk, and when done by occupational therapists)	1 [48]	• Home safety assessment and modification ✓
Knowledge interventions	1 [34]	4	5	x	0	x
Other (vibration intervention without exercise)	0	0	0	–	1 [51]	• Low magnitude/high frequency vibration (standing still on machine) ✓
Multiple interventions	3 [34, 37, 41]	15	18	Data not pooled because of heterogeneity of interventions	2 [50, 53]	• Education + geriatric clinic assessment ✓
Multiple interventions including an exercise intervention	2 [34, 37]	14	16	Separate meta-analysis for exercise related multiple interventions not conducted in the Cochrane review	2 [50, 53]	x
Multifactorial interventions	4 [35, 36, 39, 40]	36	40	RaR 0.76 (0.67–0.86) for rate of falls outcome	0	• Improved pre-operative, post-operative and post discharge management (multifactorial, multidisciplinary) for patients after hip fracture

Note – for multiple intervention studies where individual outcome data were reported for a single intervention as well as a multiple intervention arm, these are reported in both intervention types in the Table

✓ = significant reduction in fall rate or falls risk, or both; x = no significant effect on falls outcomes; ≈ = borderline significance

^#^One or more randomised trials conducted in Asia with significantly reduced falls outcome (falls, falls risk, fall injuries) – refer to Table 2 for details of individual studies

**Fig. 1:**
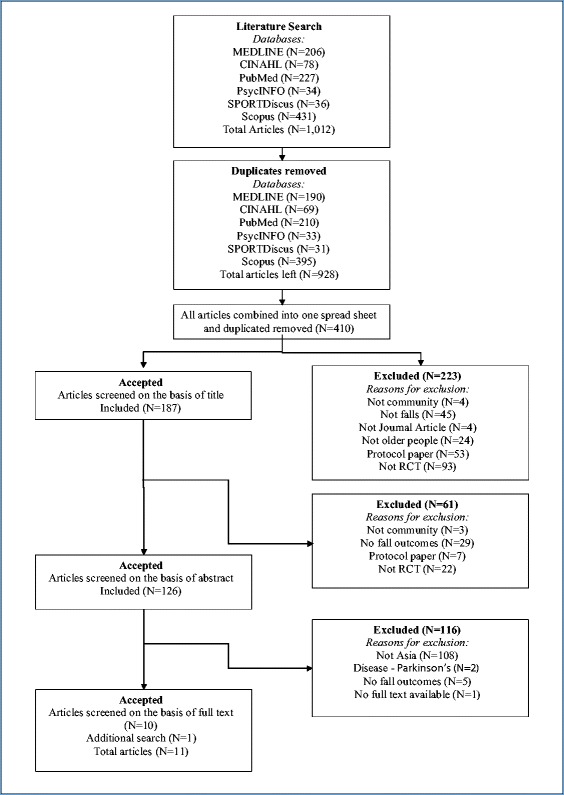
Study selection flowchart for randomised trials published after the 2012 Cochrane

An additional 11 RCTs were identified that were conducted in Asian populations, met the inclusion criteria for the community based falls prevention Cochrane review, and were published after the 2012 Cochrane review (Japan *n* = 6; [43–48] Taiwan *n* = 2; [49, 50] and China/Hong Kong, [51] Malaysia [52] and Singapore [53] *n* = 1 each). See Fig. 1 for study selection flowchart to identify papers after 2012. Seven of the additional studies published since the 2012 review were exercise interventions.

Combining the Asian studies in the 2012 Cochrane review and subsequent Asian RCTS, the only single intervention types that had more than one randomized controlled trial conducted in Asia were exercise (*n* = 15), and environment (home hazard assessment and modification, *n* = 2 [38, 48]). There were also five multiple intervention studies [34, 37, 41, 50, 53] (two with two or more single intervention arms included as separate single interventions in the single intervention analyses [34, 37]) and four multifactorial intervention studies conducted in Asia [35, 36, 39, 40].

### Samples

Overall, the sample size for the studies in Asia included in the 2012 Cochrane review were relatively small (median 150, minimum 52, maximum 1043) (Table 2), whereas for the full Cochrane review [3], the median sample size across all randomized trials irrespective of where the trial was conducted was 230 (minimum 10, maximum 9940). The more recent Asian randomised controlled trials (published since the Cochrane review) have been substantially larger than those pre-2012 (median sample size 196, minimum 68, maximum 710). The samples were mixed in terms of the age inclusion criteria, with two studies including people aged over 50 years [28, 52], five had samples aged ≥60 years [37, 39, 41, 49, 51], 14 had samples aged ≥65 years [29, 32–36, 38, 42, 44, 45, 47, 48, 50, 53], three samples were aged ≥70 years [30, 40, 46], one sample was aged ≥73 years [31], and one sample was aged ≥75 years [43]. The average age of participants across the included studies in Asia was 75.1 years, and samples were on average 71.9% female (five studies had female only samples [29–31, 46, 51]).

**Table 2:** Comparison of main characteristics of interventions shown to be effective and not effective in reducing falls (F, rate of falls) or falls risk (FR, proportion of fallers) in studies conducted in Asia

	Sample and design	Details of intervention	Control activity	Duration and frequency	Other comments	Effective (✓) or Ineffective (x) falls outcomes	Meets Sherrington’s criteria^*^
SINGLE INTERVENTIONS							
Exercise							
Suzuki et al., 2004 (Japan) ^Ø^[31]	*N* = 52; Women only. Mean age = 78; Participating in gerontology longitudinal study. RCT.	Group exercise program strength, balance, walking, Tai Chi, supplemented by home exercise program.	Pamphlet and advice on falls prevention.	Group – 10 × 1 hour session × 6 months; Home – 30 min 3 x/week.	75.3% average attendance at exercise classes. Follow-up at 8 and 20 months for falls data. 20 month data reported in the Cochrane review.	**✓** • F – RaR = 0.35 [0.14, 0.88] • FR – RR = 0.25 [0.08, 0.78] Intervention group significant improvements on tandem walking, Functional Reach and knee extension power.	Bal ✓ Dose ✓
Woo et al., 2007 (Hong Kong, China) [42]	*N* = 180 (90 men and 90 women); Mean age 69 years, range 65–74. RCT with block randomization by gender.	(1) 24 form Yang style Tai Chi (2) Resistance exercises with thera-band.	Usual care.	(1) Tai Chi 24 form, 3 x/week × 12 months (2) Resistance exercise 3 x/week 12 months (arm lifting, hip abduction, heel raise, hip flexion, hip extension, squatting ankle dorsiflexion).	High compliance – Tai Chi 81%, resistance training 76%. 12 month follow-up.	(1) ✓ (2) x • (1) FR – RR = 0.48 [0.29, 0.80]^##^ • (2) FR - 0.77 [0.52, 1.14] No significant between group differences for strength, balance or flexibility measures. Sub-group analysis in women identified significantly reduced rate of loss of total hip bone mineral density in both exercise groups relative to control group.	(1) Bal ✓ Dose ✓ (2) Bal? (not clear starting position or hand support) Dose ✓
Lin et al., 2007 (Taiwan, rural/agricultural area) [38]	*N* = 150, recent fallers. 51% female. Mean age = 76.8 years. RCT.	(1) Home exercise with physio (individualized flexibility, strength & balance exercises) (2) Home safety assessment and modification by public health worker (see below for results)	Education and social visit every 2 weeks with public health worker + falls prevention brochure.	(1) 40–60 min, 3 x/week × 4 months, physio visit every 2 weeks (2) 30–40 min visit every 2 weeks to perform safety assessment and make recommendations.	Assessment at 2 and 4 months for quality of life, depression and physical performance measures. Adherence to exercise program not reported.	x (exercise) • F – RaR = 0.67 [0.35, 1.28] Exercise group had significant improvements on Functional Reach, Tinetti POMA, and fear of falling than the Education group.	(1) Home exercise program Bal ✓ Dose x
Shigematsu et al., 2008 (Japan) [32]	*N* = 68. 63% female. Mean age 69 years, range 65–74. RCT.	Square stepping exercise, included forward, backward, lateral, and oblique stepping patterns on a thin felt mat, added challenge after familiarity by walking on toes; and increased complexity of step pattern.	Supervised outdoor walking program - 40 min, 1 x/week × 12 weeks, emphasis on increasing steps.	70 min, 2 x/week × 12 weeks.	Adherence: Square stepping exercise – 91% of sessions, outdoor walking – 84% of sessions. Falls data followed up for 8 months.	x • F – RaR = 0.70 [0.23, 2.13] • FR – RR = 0.64 [0.21, 1.95] Significant improvement in the Square stepping exercise group on leg extension power, forward/backward tandem walking, stepping with both feet, simple/choice reaction time, and perceived health status.	Bal ✓ Dose x
Iwamoto et al., 2009 (Japan) Ø [28]	N = 68. Attending orthopaedic clinics. 90% female. Age > 50 (mean age = 76.4 years). RCT.	Calisthenics, balance, power and walking exercises (home based, but 3 x/week supervision in clinic).	Usual care.	Daily exercise, with supervision in clinic 3 x/week × 30 min. Duration of exercise 5 months.	Exercise adherence reported as 100%.	x • FR – RR = 0.11 [0.01, 1.52]^#^ Study reports significant reduction in falls, but effect not significant in Cochrane review. Significant improvement in flexibility, balance, mobility and gait measures in the exercise group relative to the control group.	Bal ✓ Dose x
Kamide et al., 2009 (Japan) Ø [29]	*N* = 57. 100% female. Attending employment agency (for light work or volunteer activity). Age > 65 (mean age = 71 years). RCT.	1 × 1 h education session (focus on osteoporosis fracture prevention and exercise) and 1 h training for home exercise; then home based exercise program (flexibility, balance, strength and impact exercises).	Usual care. Therapist contact by phone or mail each 3 months.	3 days/week × 6 months. No home visits by therapist re exercise program, but contact by phone or mail monthly to support motivation.	Exercise adherence - 82% of exercise participants completed the study. Of these, 91% performed exercises at least twice weekly. Follow-up over 12 months.	x • FR – RR = 0.38 [0.02, 7.91] Significant improvement in Timed Up and Go in exercise group relative to the control group.	Bal ✓ Dose ✓
Yamada et al., 2010 (Japan) Ø [33]	*N* = 60. % female not stated. Age > 65 (mean age approx 80 years). RCT.	Exercise class + multi-component trail walking program. Variability in how program was implemented to add challenge and motivation.	Exercise class + simple indoor walking program.	Exercise class 1 x/week × 16 weeks (60 min, included aerobic, strength, balance and flexibility exercises). Trail walking involved walking to set flags in order, changing direction, focus on speed. 30 min/session. Indoor walking program was 30 min/session.	Adherence – median for both group 100%	x • F – RaR = 0.45 [0.14, 1.49] • FR – RR = 0.45 [0.18, 1.13] Study reported reduced falls at 6 months, not sustained at 12 months. Cochrane review utilized 12 months falls data. Significant improvement in Timed Up and Go, walking task, and dual task gait tasks for Trail walking group relative to indoor walking group.	Trail walking program Bal ✓ Dose x NB – both groups received multimodal exercise class.
Huang et al., 2010 (Taiwan) Ø [34]	*N* = 261 randomized, *N* = 163 follow-up. 48% female after loss to follow-up. Age > 65 (mean age = 71 years). Cluster RCT (by village). 5 month intervention and 12 month post intervention follow-up.	(1) Education (falls risk factors, and reducing falls risk) (2) Tai Chi (3) Combined education + Tai Chi as above.	Not described.	(1) 5 × 1 h group sessions across 5 months. (2) 40 min sessions, 3 x/week × 5 months. (3) Combined education and Tai Chi program.	18 month follow-up for falls data. High drop-out rates over 5 month intervention period – education (52%), Tai Chi (52%), Education + Tai Chi (34%), control (6%).	(2) x • FR – RR = 0.51 [0.02, 12.49] Cochrane review used raw data at 5 months only, as 18 month raw data not provided. Cochrane review reports all interventions as non-significant (although combined education and Tai Chi reported as effective in reducing falls at 5 months, and all three interventions as effective in reducing falls at 18 months in the paper). Secondary measures only compared pre –post (within group).	(2) Tai Chi Bal ✓ Dose x
Yamada et al. 2012 (Japan) Ø [43]	*N* = 157. 87.8% female. Age ≥ 75 years (mean age 85.5 years).	All participants (intervention and control groups) received 45 min of group training sessions 1 x weekly (strength, balance, cardiovascular, flexibility exercises). In addition, the intervention group undertook a complex obstacle course negotiation program each session (finding marker to walk to, direction changes, avoiding obstacles).	Same main exercise class as intervention group, but undertook an additional simple obstacle course negotiation program (6 trials / session of 15 m walkway with obstacles interspersed along walkway).	24 weeks, once weekly sessions. Two trials of finding 15 markers/session in addition to common exercise program once weekly, 45 min duration).	Median adherence in both groups – 96%. No significant difference between groups on balance and mobility measures after intervention (except for a complex obstacle negotiation task, with the Intervention group achieving significantly greater improvement than the Control group).	✓ Fallers – 2 in intervention group (2.8%), and 19 (26.0%) in the control (simple obstacle course group). IRR for falls in the control group relative to intervention group was 9.37 (2.26– 38.77). IRR for fall-related fractures in the control group relative to intervention group was 7.89 (1.01–61.49).	Bal ✓ Dose x (comparing difference in exercise time between two groups)
Yamada et al., 2013 (Japan) Ø [44]	*N* = 264. 57.3% female. Age ≥ 65 years (mean age 76.7 years).	All participants (Intervention and Control groups) received 30 min of group training session (2 x weekly × 30 min, aerobic, strength, balance and flexibility exercises). In addition, the Intervention group undertook a Multi-task Stepping activity each session, that involved varied stepping pattern along a walkway, at comfortable speed.	Same main exercise class as intervention group, but undertook an additional	Twice weekly for 24 weeks. Total time spent walking on the mat during the Multi-task Stepping Intervention was 1 to 2 min/task, repeated 4 times/session (total additional time of Multi-task Stepping/session was 5 to 7 min).	Intervention group achieved significant improvement relative to Control group in walk time and Timed Up and Go.	✓ Fallers: 13 Intervention participants (11.6%) and 39 (33.0%) in the Control group fell during the 12-month follow-up period. IRR for falls in the Intervention group relative to the Control group was 0.35 (0.19–0.66). Fall-related fractures: 3 participants in the Intervention group had fall related fractures compared to 13 participants in the Control group RR for fall-related fractures in the Intervention group relative to the Control group was 0.22 (0.06–0.80).	Bal ✓ Dose x
Ohtake et al. 2013 (Japan) [45]	*N* = 196. 83.5% female. Age > 65 years (mean age = 83.6 years).	Both intervention and control group received a health education program on falls prevention. Intervention group also undertook a group based exercise program (strength, balance and flexibility).	Health education program on falls prevention (same program also delivered to intervention group)	8 week exercise program once weekly (20–30 min), together with 1–2 x weekly home exercise program	8.9% of the exercise group dropped out after baseline assessment. 97% participation rate in the group exercise sessions, participants also did on average 3.8 days/week of home exercise.	x Small number of fallers in each group – Intervention group n = 7 (7.6%); and in the control group *n* = 9 (12.1%) (*p* = 0.323). Significant improvement in Functional Reach and falls efficacy in the intervention group relative to control group.	Bal ✓ Dose x
Kim et al., 2014 (Japan) Ø [46]	*N* = 105. 100% female. Age > 70 years (mean age = 77.8 years). Participants had one or more falls in the past year.	Group based strength and balance exercise program.	3 month health education sessions (60 mins each month).	3 month group program twice weekly × 60 min, then 4–12 months 1 x monthly group exercise program supplemented with home exercise ≥3 times weekly.		✓ At 12 months: Falls – I 19.6%, C 40.4%, (OR 2.78, 1.17–6.96); Repeated falls - I 20%, C 33.3%, (OR 1.85, 0.33–7.38); Injurious falls – I 80%, C 62%, (OR 0.82, 0.22–3.05). The exercise group significantly improved in one leg standing time, knee extension strength and ankle dorsiflexion strength over the 12 months, compared to the education and excluded group who showed no significant improvements.	Bal ✓ Dose ✓
Hirase et al., 2015 (Japan) Ø [47]	*N* = 93 older adults using community day centers. 69.9% female. Age > 65 years (mean age 82.1 years)	Group based programs: (1) Foam rubber balance exercises (2) Stable surface exercises. Both interventions were supplemented with a daily home exercise program (2–3 exercises).	Continued activities at the day centres, but did not perform balance or strengthening exercises.	4 months program, Once weekly 60 min exercise class supplemented with home exercise program (for both intervention groups).	7.5% of participants withdrew from the study. High adherence to the exercise programs: 95.5%, 93.3% of all possible classes in the foam rubber and stable surface groups.	x Mean number (SD) of falls for the foam rubber, stable surface, and control groups was 0.24 (0.51), 0.59 (1.94), and 0.90 (1.45) (*p* = 0.07). Significant improvement for both exercise groups compared to the control group for balance and sit to stand measures. Significant improvement for foam exercise group compared to firm surface exercise group for some balance tests at 2, 3 and 4 months.	Bal ✓ Dose ✓
Ashari et al., 2016 (Malaysia) [52]	N = 68. 57.4% female. Age > 50 (mean age = 63.7 years) Inclusion criteria of impaired turning performance (Neurocom Force Platform)	Individualised home based exercise program, based on Otago Exercise Program. 6–8 balance and strengthening exercises (including 2 turning exercises).	Maintain usual activities	16 week program, 20–30 min/day, ≥4 times/week, and walking program ≥3 times / week.	91% I group completed 16 week program	x Fallers in 16 week program – I 5.8%, C 8.8% (underpowered, no significance testing). I significantly improved relative to C on turning measures, Timed Up and Go (single and dual task) and static stance sway.	Bal ✓ Dose ✓
Hwang et al., 2016 (Taiwan) Ø [49]	*N* = 456. 67% female. Age ≥ 60 years (mean age = 72.4 years). Participants had one or more Emergency Dept presentation due to a fall 6 or more months prior to study.	Compared two 24 week home-based exercise interventions: (1) Yang style Tai Chi (2) Lower Extremity Training (LET - balance, strength and flexibility exercises, individualised) Both groups had one supervised session/week and were encouraged to self practice daily at home. After 6 months (when supervised exercise ceased) participants asked to continue self practice daily.	No control group	(1) Tai Chi - 60 min supervised session (10-min warm-up followed by a review of previous movements, introduction of new movements, and 5 min of relaxation) (2) Lower Extremity Training - 60 min physio supervised session (10-min warm-up, 45 min of exercise, and a 5-min cool-down).	Results reported for 6 month intervention period, and subsequent 12 months. Adherence: 78% of Tai Chi participants and 72% of the LET group participated in ≥20 of the 24 (83%) sessions. During the 6-month intervention, 50% of the Tai Chi participants and 67% of the LET group independently practiced the exercise program ≥7 times per week.	✓ At 6 months-falls: F: IR (Tai Chi/LET) 0.30 (0.15–0.60) FR: RR (Tai Chi/LET) 0.76 (0.66–0.87). At 6 months - Injurious falls: F: IR (Tai Chi/LET) 0.33 (0.16–0.68) FR: RR (Tai Chi/LET) 0.86 (0.77–0.96). At 18 months: falls and injurious falls remained significantly reduced for the Tai Chi group compared to the LET group. For the Tai Chi group, handgrip strength, Tinetti balance and gait, depression, and cognition scores improved significantly during the 6-month intervention. For the Lower Extremity Training group, handgrip strength, Tinetti balance and gait, fear of falling, depression, and cognition scores improved significantly during the 6-month intervention.	Bal ✓ Dose ✓
Medication							
Sato et al., 2005 (Japan) [30]	*N* = 200. 100% female. Age > 70 years (mean age = 78 years). Ambulatory women recruited from an out-patient department with probable Alzheimer’s disease. RCT.	45 mg menatetrenone (vitamin K2), 1000 IU ergocalciferol (vitamin D2), and 600 mg calcium.	Usual care.	Daily medication for 2 years.	Significant reduction in fractures.	**✓** • FR – RR = 0.13 [0.04, 0.43]	
Psychological intervention							
Huang et al., 2011 (Taiwan) Ø [37]	*N* = 186. 59% female Age ≥ 60 years. RCT.	(1) Cognitive behavior therapy group, intervention based on previous program, [69] but added newly developed fear of falling management model developed by first author. (2) Cognitive behavior therapy group + Tai Chi	Usual care.	(1) 60–90 min weekly × 8 weeks (2) CBT as above + Tai Chi 60 min 5 x/week × 8 weeks.	Outcomes assessed at 2 and 5 months. NOTE: Tai Chi group total exercise dosage 40 h.	**x** • F – RaR = 1.00 [0.37, 2.72] • FR – RR = 1.00 [0.40, 2.51]	
Environment/assistive technology intervention							
Lin et al., 2007 (Taiwan, rural/agricultural area) [38]	See above.	(1) Home exercise with physio (individualized flexibility, strength & balance exercises) (see above for outcomes) (2) Home Safety Assessment and Modification by public health worker.	See above.		For the Home Assessment and Modification intervention, 14 inexpensive modifications (of a list of 28 options) were implemented within the first week of the intervention. Other recommended modifications were recommended to the family by the assessor (a public health worker). No data provided on adherence to home modifications provided, nor for uptake of the additional recommended home modifications.	(2) ✓(Home modification)^##^ • F – RaR = 0.46 [0.22, 0.95] No significant differences for the Home Assessment and Modification group on the WHOQOL-BREF domains relative to the Education group.	
Kamei et al., 2015 (Japan) Ø [48]	*N* = 130. 84.6% female. Age > 65 years (mean age 75.8 years).	Both groups undertook the same 4 x weekly falls prevention multifactorial Program (120 min each) covering physical and mental assessment interview; (ii) blood pressure check; (iii) education regarding fall risk factors, food and nutrition, foot self-care; and (iv) exercise sessions focussed on strength, coordination and balance. Intervention group also received a home hazard checklist, a training program on home hazard awareness and modification	4 weekly multifactorial program as described for intervention group.	4 weeks intervention, 120 min/session.	16.4% of the intervention group did not attend sessions regularly and withdrew from the study. Intervention group significantly improved falls prevention awareness and home modifications.	x 10.9% reduction in all falls in intervention group compared to control group. Time to first fall: HR = 0.591 (0.305–1.147), *p* = 0.116. Indoor falls – reduced by 11.7% in Intervention group relative to control group HR = 0.397 (0.151–1.045), *p* = 0.052.	
Knowledge intervention							
Huang et al., 2010 (Taiwan) Ø [34]	See above.	Education intervention included separate sessions on medications, nutrition, safe home environment, and footwear. It included a component of each session for revision.				x • FR – RR = 1.62 [0.11, 24.16] The Education group achieved improved score post relative to pre intervention on indoor environment score, fear of falling, and Timed Up and Go. Significance of differences in change between groups with the intervention relative to control was not provided.	
Other							
Leung et al., 2014 (China) Ø [51] NOTE: Some vibration studies incorporate exercise during vibration – this intervention involved participants standing with knees straight – no exercise	*N* = 710. 100% female (post-menopausal). Aged >60 years (mean age 72.9 years). Cluster RCT. Participants recruited through community centres for older people.	Low magnitude high frequency vibration – standing upright without knee bending on a purpose built vibration platform that provided vertical synchronous vibration at 35 Hz, 0.3 g.	Habitual lifestyle, participated in normal interest group activities run by the community centres.	18 months, 5 x/week × 20 min standing on vibration platform.	29.7% of vibration group were lost to follow-up at 18 months (most of these declined to continue participation). No serious adverse events, though nine vibration participants and seven control participants complained of leg pain; and five vibration and one control participant complained of dizziness.	**✓** Fall or fracture incidence: I – 18.6% C – 28.7%. Adjusted Incident Rate Ratio for falls or fractures: 0.54, 95%CI 0.37–0.78, *p* = 0.001. Significant improvement for vibration group on secondary measures including leg muscle strength and balance.	
Multiple interventions							
Assantachai et al., 2002 (Thailand) Ø [41]	*N* = 1043. 64% female. Age > 60 (mean age = 68). Cluster RCT (by community).	Received information leaflet describing risk factors for falls and strategies to reduce risk. Risk factors covered included nutritional advice (including calcium intake), activities of daily living, hypertension, special sense function and high risk medications. Also offered free access to geriatric clinic for any health problem patients wanted reviewed.	Usual care	No information provided regarding the proportion of the intervention group who took up the offer of free access to the geriatric clinic, what type of interventions were provided for those who accessed it, and their adherence.		**✓** • FR – RR = 0.77 [0.63, 0.94]	
Huang et al., 2010 (Taiwan) Ø [34]	See above (Study had three intervention groups and control – Education only, Tai Chi only and combined Tai Chi and Education).	Combined program incorporated 5 x education sessions over 5 months and a Tai Chi (13 forms) exercise program – 40 min/session, 3 x/week for 5 months.			High drop-out rates over 5 month intervention period - Education + Tai Chi group (34%). NOTE: Tai Chi group total exercise dosage 40 h.	x • FR – RR = 1.68 [0.16, 17.67] Cochrane review used raw data at 5 months only, as 18 month raw data not provided. Cochrane review reports all interventions as non-significant (although combined education and Tai Chi group reported as reducing falls at 5 and 18 months in the paper).	
Huang et al., 2011 (Taiwan) Ø [37]	See above (Cognitive Behavioral Therapy + Tai Chi) (also compared to Cognitive Behavior Therapy alone)	Cognitive Behavioral Therapy program based on previous program, [69] but added newly developed fear of falling management model developed by first author. This arm of the intervention combined the cognitive behavior therapy with a Tai Chi exercise program.	Usual care.	Cognitive Behavior Therapy (CBT) and Tai Chi combined program incorporated 60–90 min weekly × 8 weeks for the CBT and 60 min 5 x / week × 8 weeks for the Tai Chi component.	Outcomes assessed at 2 and 5 months. NOTE: Tai Chi group total exercise dosage 40 h.	x • F – RaR = 0.38 [0.10, 1.47] • FR – RR = 0.40 [0.11, 1.45] The combined Cognitive Behavior Therapy + Tai Chi group achieved significantly improved falls efficacy, improved mobility, higher social support satisfaction, and quality of life than the control or cognitive behavior therapy alone groups.	
Lee et al., 2013 (Taiwan) Ø [50]	*N* = 616. 55% female. Age > 65 years (mean age 75.7 years). Participants had high falls risk, with any of: (1) recurrent falls in the previous year; (2) medical history associated with high falls risk (ie, stroke, Parkinson’s disease, head injury, fractures due to falls); and (3) fell only once in the previous year, and had gait or balance problems (poor TUG score)	All intervention participants received: 1. Strength, balance, cardiovascular and flexibility group exercise program, supplemented with home exercise program. 2. Health education sessions and brochures on falls prevention. 3. Home hazards evaluation/modification. 4. Medication review 5. Ophthalmology/other specialty consults.	Health education brochures, medication reviews and medical referrals without direct exercise interventions or structured health education sessions.	3 month multifactorial intervention	Attrition rate for 3 month intervention period: I 10.9%; C 13.5%.	x For 12 month followup period: F: I 128 (0.41 falls/person year), C 132 (0.44 falls/person year) (*p* = 0.692) FR: HR 0.90 (0.66–1.23). At end of 3 month intervention: Significant improvement in intervention group relative to control group for Physiological Profile Assessment, reaction time, balance, mobility and depression (although control group improved significantly more than intervention group on knee strength and proprioception).	
Ng et al., 2015 (Singapore) [53]	*N* = 246. 61% female. Age > 65 years (mean age 70). Factorial design RCT, with nutrition, exercise and cognitive training groups, and combined intervention group. Recruited frail and pre-frail older people based Cardiovascular Health Study (frailty phenotype) criteria.	Nutrition group: nutritional supplementation with commercial formula, iron and folate, Vit B6 and B12, calcium and vitamin D supplements daily. Exercise: Strength and balance group program (weeks 1–12) then home program weeks 13–24. Cognitive training: Cognitive enhancing activities including verbal recall, mazes, problem solving etc. Combination group: all of the above interventions	Standard care + placebo supplement liquid + placebo capsule and tablets (identical appearance to intervention nutrition supplements), and instructions not to replace their meals with the supplements.	6 month intervention period. Nutrition: supplements daily. Exercise: Weeks 1–12, twice weekly group sessions; weeks 13–24 home exercise 2 h weekly. Cognitive training: Weeks 1–12 - 2 h/week; weeks 13–24 – fortnightly 2 h booster sessions.	Low dropout rate (8% for nutritional supplement; 10% cognitive training; 4% for exercise; 6% for combination, and 8% for control).	x Small numbers of fallers/group, analysis only provided across all groups (12 months, *p* = 0.67). Fallers/group (12 months): Nutrition: 4 fallers (8.6%) Exercise: 3 fallers (6.3%) Cognitive training: 2 fallers (4.1%) Combination: 2 fallers (4.1%) Control: 5 fallers (10.4%)	
Multifactorial interventions							
Jitapunkul et al., 1998 (Thailand) [40]	*N* = 160. 65% female. Age ≥ 70 years (mean age = 76 years). Participants recruited from RCT.	Home visit by non-professional with a structured health questionnaire. Referral to a nurse or geriatrician if function declined or ≥1 fall in 3 months, with subsequent nurse or geriatrician home visit to assess, educate, prescribe, or make other referrals.	Usual care. Assessment at end of 3 year period.	Home visit at study commencement, then three monthly visits × 3 years.	Intervention group had significantly less rate of functional decline (Chula ADL Index and Barthel ADL Index) over the study period.	x • FR – RR = 0.52 [0.14, 1.94]	
Huang and Acton, 2004 (Taiwan) Ø [35]	*N* = 120. 46% female. Age ≥ 65 years (mean age = 72 years). RCT.	Falls prevention information brochure + individualized falls prevention information (medication and home safety focus) – brochure and verbal.	Falls prevention information brochure.	Three home visits by nurse in 4 months (a) for initial assessment), (b) to work through individualized risk factors (medication and home safety), and (c) re-assessment at 4 months.		x • FR – RR = 0.12 [0.01, 1.76] Improved knowledge of medications and reduced home hazards in intervention group. Only 2 months follow-up after intervention provided.	
Huang et al., 2005 (Taiwan) Ø [36]	*N* = 141. 69% female. Age > 65 years (mean age = 77 years). Hip fracture patients recruited and randomized at hospital discharge. RCT. Patients interviewed at discharge, 2 weeks post-discharge and 3 months post-discharge.	Enhanced discharge planning by experienced gerontological nurse, including visits on wards, home visit, and phone contacts post discharge. Included discharge and falls prevention brochure. Involved patient, family and other health care professionals. Included nurse follow-up with physicians.	Usual discharge planning (no brochures, no written discharge summaries, no home visit, no telephone contact).	Visits on wards at least every 2 days, home visit within 3–7 days of discharge, and once weekly phone calls post discharge.	Positive outcomes for the intervention group included significantly reduced hospital length of stay.	x • FR – RR = 0.67 [0.22, 2.01]	
Shyu et al., 2010 (Taiwan) Ø [39]	*N* = 162. 69% female. Age ≥ 60 years (mean age = 78 years). Patients admitted to hospital for hip arthroplasty or internal fixation. RCT. Excluded patients with cognitive impairment, marked functional impairment pre-operatively, or those who were terminally ill.	Three components to intervention: (1) geriatrician review; (2) rehabilitation; and (3) discharge planning service.	Usual care, described as limited interdisciplinary involvement, usually no home visit, and no in-home physiotherapy.	(1) Geriatrician/geriatric nurse review and recommendations pre-operatively, and geriatric nurse review and recommendations post-operatively. (2) Focus on early post-operative rehabilitation, and in-home rehabilitation. (3) Discharge planning was coordinated by a geriatric nurse, and included a pre-discharge home visit and recommendations, and follow-up phone calls. On average, there was 1 x geriatrician visit, 5.4 geriatric nurse visits, 3.1 physical therapist visits, and 1 rehabilitation physician visit during hospitalization; and 9.9 geriatric nurse and 3.0 physical therapist home visits after return home.	Intervention group had significant improvement relative to control group on Activities of Daily Living, walking ability, reduced depression and better SF36 scores (two year follow-up).	**✓** • FR – RR = 0.56 [0.34, 0.93]	

NB: for multiple intervention studies where results have been reported against a control group for individual interventions, these have been included in the single intervention component of the table as well

RaR = Rate Ratio; RR = Risk Ratio

✓=yes; x= no

* Criteria based on Sherrington's review and meta-analysis [54] (for exercise studies only: (1) moderate to high challenge to balance; and (2) at least 50 h total dosage

^#^Reported as non-significant falls outcome in Cochrane review, although paper reported significant reduction in incidence of falls

^##^Reported as non significant falls outcome in the published paper, however reported as significant reduction in falls outcome/s in the Cochrane review

Ø Indicates that study was conducted with a primary focus on prevention of falls (identified in aim, hypothesis, or as primary outcome / used for power calculation)

POMA = Problem Oriented Mobility Assessment; RCT = Randomized Controlled Trial; Bal = Balance; IU=International Units; CBT = Cognitive Behavioral Therapy; ADL = Activities of Daily Living

### Intervention types

Table 1 reports details of individual intervention types conducted in Asia relative to the rest of the world, while Table 2 summarizes the main characteristics of the trials conducted in Asia, grouped by intervention type, including details of whether or not the intervention was effective in reducing the rate of falls or number of people falling.

### Single interventions

#### Exercise interventions

Fifteen trials from Asia reported results for an exercise intervention as a single intervention, with only six reporting one or more positive fall related outcomes [31, 42–44, 46, 49]. The exercise trials generally had small sample sizes (60% *n* < 70, median sample size *n* = 105). Three of the effective exercise programs used Tai Chi (20% of all exercise trials) either as the sole intervention [42, 49] or combined with other balance and strength exercises [31]. In contrast, only five of the 51 (10%) exercise trials from the 2012 Cochrane review conducted outside of Asia involved Tai Chi. The other effective exercise approaches in Asian studies used an obstacle course [43], a multi-target stepping program [44], and a group balance and strength training program [46]. Only one of the ineffective exercise trials used Tai Chi, [34] other ineffective programs used home programs, [29, 38, 52] group programs (balance [47] or multimodal [45]), a group exercise program combined with trail walking, [33] combined group and home based program, [28] and a square stepping program [32]. Effective exercise programs ranged from 24 to 52 weeks duration, and had from 1 to 8 times per week of recommended exercise (included both supervised and home-based sessions).

In a separate review, Sherrington and colleagues reviewed 54 randomized controlled trials evaluating exercise interventions to reduce falls, and identified several key criteria that appeared to differentiate effective from ineffective falls prevention trials [54]. Two of the key criteria related to: (1) the exercise intervention having a moderate to high challenge to balance; and (2) a minimum of 50 h overall exercise dosage. Of the six effective exercise trials in Asia, four (67%) met both of these criteria [31, 42, 46, 49]. The other two effective exercise studies [43, 44] met the “challenge to balance” criteria, but involved substantially less than the 50 h duration. Three of the ineffective exercise studies met both of Sherrington’s criteria, but were likely to be underpowered (sample sizes of 57 [29], 68 [52] and 93 [47]). Most of the remaining ineffective exercise interventions did not meet the dosage criteria, although the majority met the balance challenge criteria. Although adherence to the exercise programs was reported in ten of the studies, the method of reporting was variable, making comparisons difficult. The study by Huang and colleagues reported high attrition in the Tai Chi group (52%) over the five month intervention [34]. All except two of the studies reported significant improvements in the secondary balance, strength, or mobility related measures in the exercise intervention relative to the control group [34, 42].

#### Medication interventions

Medication interventions can include approaches to review or reduce medications overall, or specifically target high risk medications such as psychotropic medications; or provide supplementation to improve fall related outcomes [3]. Only one Asian study investigated a medication related intervention, by evaluating the effect of two years supplementation with vitamin K2, vitamin D2 and calcium, compared to usual care, in older women with probable Alzheimer’s disease [30]. There was no effect on falls outcomes, but a significant reduction in fractures in the medication intervention group.

#### Psychological interventions

One study investigated the effectiveness of Cognitive Behavioral Therapy (CBT) alone, and in combination with Tai Chi, relative to a control group receiving usual care [37]. Neither CBT alone or combined with Tai Chi reduced falls outcomes. CBT alone did not result in significant improvements in any secondary measures, including falls efficacy, relative to the control group (see multiple interventions for outcomes for the combined Tai Chi and CBT intervention group).

#### Environment/assistive technology interventions

Two studies investigated home assessment and modification interventions. In a Japanese study, Kamei et al. evaluated the effect of a home hazard checklist and a training program on home hazard awareness for older people, superimposed on a multifactorial assessment and intervention program received by both the intervention and control group (both groups received a falls risk factor education program, exercise, blood pressure review, and physical and cognitive assessments) [48]. Although the intervention group significantly increased their falls prevention awareness and home modifications implemented, there was no significant reduction in falls. Another randomized trial in Taiwan compared a home assessment and modification intervention against an education intervention, and an exercise intervention [38]. The home modification program was conducted by a public health worker, and involved a standard assessment, provision of 14 standard, inexpensive modifications (eg removal of loose mats, marking of step edges, rectification of poor lighting), and recommendations regarding another 14 modifications if required. Although the home safety assessment and modification intervention was reported by Lin et al. [38] as not achieving a significant reduction in rate of falls, the reduction was significant in the Cochrane review analysis [3]. There was no information provided about the range of additional recommendations made, nor the level of adherence with the implemented or recommended home modifications.

#### Knowledge interventions

One randomized trial from Taiwan investigated the effect of knowledge based interventions provided to older people on reducing risk of falls [34]. The education intervention involved five one hour group education sessions over five months, targeting separate important risk factors at each session [34]. While the intervention achieved improved knowledge about falls risk, there was no reduction in falls outcomes.

#### Other single interventions

There were a number of additional interventions that have been shown to be effective in reducing falls, falls risk or falls injuries in the 2012 falls prevention in the community setting Cochrane review, which have not been investigated in any randomized controlled trials in Asian countries [3]. These include (a) medication prescription review; and high-risk medication withdrawal (eg psychotropic medications); (b) cataract surgery (first eye); (c) changing from bifocal or multifocal glasses to distance glasses for outdoors mobility; (d) cardiac pacing surgery (for carotid artery hypersensitivity); and (e) footwear (anti-slip shoe device for icy conditions). Vitamin D supplementation, which was considered effective in reducing falls in at-risk populations by the Cochrane review, has also not been evaluated for its effect on falls in Asian populations as a single vitamin supplement (Sato and colleagues evaluated supplementation of vitamin K2, vitamin D2 and calcium as a single intervention) [30]. Some of these interventions may be inappropriate in many parts of Asia (eg anti-slip shoe device for icy conditions). However, the other intervention types are likely to have direct or perhaps modified applicability for older people in Asia.

One recent trial in Asia has been classified as “Other” under the single intervention studies – an 18 month investigation of low-magnitude high-frequency vibration program in post-menopausal women in China/Hong Kong (*n* = 710) [51]. Vibration interventions often utilize exercises while performing vibration, [55] and so may be classified under exercise interventions, however the study by Leung and colleagues had participants standing with straight knees on the vibrating platform for the duration of the vibration procedure (20 min/session). The group receiving the vibration therapy had significantly reduced Hazard Ratio for the combined outcome of “falls or fractures” ([0.56, 0.40–0.78, *p* = 0.001).

### Multiple interventions

Five randomized controlled trials evaluated the effect of a multiple intervention approach to reducing falls [34, 37, 41, 50, 53]. Only one of the trials was effective, providing education (brochure targeting a number of important falls risk factors) and free access to a geriatric clinic as required for the intervention group [41]. Although effective, no details were provided about the uptake, type of interventions, or adherence to recommended interventions for the intervention group. The ineffective interventions included a combined Tai Chi exercise program and education program (each of these two components were also evaluated in isolation); [34] a combined Tai Chi and Cognitive Behavioral Therapy program; [37] a strength, balance, and fitness exercise program combined with health education, home assessment and modification, medication review, and ophthalmology or other specialty consultations; [50] and a factorial design study with participants receiving one or more of nutrition, exercise, and cognitive training interventions [53]. Tai Chi was shown to be an effective exercise intervention to reduce falls when used as a single intervention in three Asian studies, [31, 42, 49] and in non-Asian countries [3]. In both of the ineffective multiple intervention studies incorporating Tai Chi, the Tai Chi component did not incorporate the 50 h of exercise recommended to improve likelihood of achieving a significant reduction in falls [54].

### Multifactorial interventions

Four of the randomized controlled trials conducted in Asia utilized a multifactorial falls prevention intervention [35, 36, 39, 40]. Two of these targeted a high-risk population (patients with hip fracture returning home after surgery), and utilized improved discharge planning and post discharge follow-up, including home visit/s [36, 39]. The study by Shyu and colleagues also incorporated a strong interdisciplinary care model pre and post-surgery [39]. The trial conducted by Jitapunkul and colleagues utilized a regular (three monthly) home visit health screening process by a non-health professional, with subsequent referral to a nurse or geriatrician if there was recent functional decline or ≥1 fall in the preceding three months. Similarly, the study by Huang and Acton utilized a targeted falls risk brochure, followed up by targeted risk factor management support by a visiting nurse (focussing on medication and home safety) [35]. Only the trial by Shyu and colleagues was effective in reducing falls in the intervention group [39].

### Quality of the studies

Risk of bias was reported for all studies published in the 2012 Cochrane review. The Cochrane Collaboration’s tool for assessing the risk of bias was used to evaluate quality of the RCTs published since Gillespie et al.’s Cochrane review. Most of the recent studies had low to medium risk of bias (Table 3). Two studies had low risk of bias across all domains, [49, 52] with all the other studies having at least one section that was unclear (authors did not provide enough evidence). The only high risk of bias was for sequence generation and blinding in the study by Ohtake et al. [45] because participants were categorized by the day of the week and they did not blind the participants, personnel or outcome assessors. The risk of bias for the studies was viewed as low to medium due to sections of data in a number of the categories not being available, and are known to be essential for conducting high quality RCTs (blinding, allocation concealment).

**Table 3:** Quality of the studies

Study	Sequence generation	Allocation concealment	Blinding	Incomplete outcome data	Selective outcome reporting	Free of other bias
Ashari et al. 2016	+	+	+	+	+	+
Hirase et al. 2015	?	+	?	+	+	?
Hwang et al. 2016	+	+	+	+	+	+
Kamai et al. 2015	?	?	?	+	+	?
Kim et al. 2014	+	?	+	+	+	?
Lee et al. 2013	+	+	?	+	+	?
Leung et al. 2014	+	+	+	+	+	?
Ng et al. 2015	+	+	+	+	+	?
Ohtake et al. 2013	–	?	–	?	+	?
Yamada et al. 2012	+	+	?	?	+	?
Yamada et al. 2013	?	?	+	?	+	?

Note. Bias was scored as low risk (+), or high risk (−) or unclear (?). [26]

### Meta-analyses

**Fig. 2:**
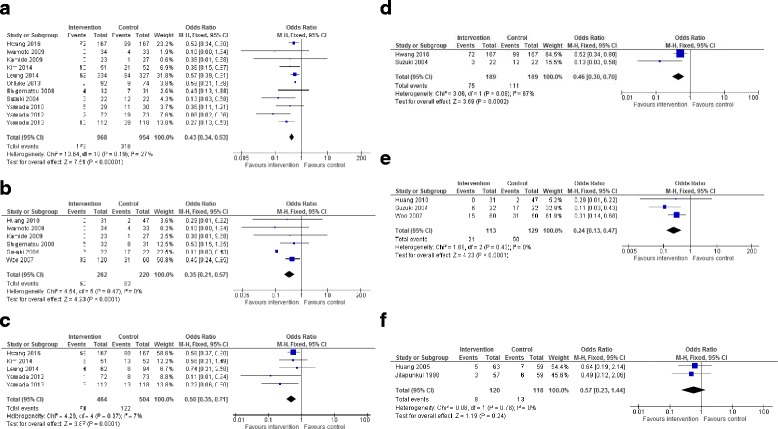
Meta-analyses of intervention types that included two or more interventions from the Asian region. **a**. Number of fallers – Exercise, **b**. Number of falls – Exercise, **c**. Number of fallers injured - exercise, **d**. Number of fallers – exercise using Tai Chi, **e**. Number of falls – exercise using Tai Chi, **f**. Number of fallers – Multifactorial Interventions

For intervention types with two or more studies from the Asian region with comparable data available, a meta-analysis was conducted. Only the exercise (*n* = 15) intervention of the single interventions, and the multifactorial interventions (*n* = 4) were included in the meta-analyses (Fig. 2). The Ashari et al. study [52] was not included in the exercise meta-analysis due to significant differences between the groups at baseline. Woo and colleagues [42] had two intervention groups and therefore dichotomous (outcome and sample) data for the intervention groups only were combined, as described in the methods. Results from the meta-analysis indicate that exercise achieved significant reduction in number of fallers (OR: 0.43 [0.34–0.53]), number of falls (OR: 0.35 [0.21–0.57]) and number of fallers injured (OR: 0.50 [0.35–0.71]). Heterogeneity was at appropriate levels for the exercise intervention meta-analyses.

A separate subgroup meta-analysis was conducted for the Tai Chi exercise interventions. Results indicated Tai Chi achieved significant reduction in the number of falls (OR: 0.24 [0.13–0.47]) and number of fallers (OR: 0.46 [0.30–0.70]), although it must be noted that there was high heterogeneity for the number of fallers (I^2^ = 67%) and therefore these results should be used with caution. Separate subgroup analysis was not able to be undertaken for the two home exercise program studies due to the Ashari and colleagues study [52] having differences at baseline discussed previously. No other sub-group analyses were possible.

The meta-analysis of the multifactorial interventions did not reach significance for number of fallers (OR: 0.57 [0.23–1.44]). Although there were two home assessment and medication trials, they reported different falls outcome data, so meta-analysis was not able to be performed. Similarly, for the falls risk outcome for the multifactorial intervention classification, there were not two studies reporting this outcome to allow meta-analysis. Similar to the Cochrane review, meta-analysis of the multiple interventions was not possible because of the diversity of the intervention types combined as multiple interventions.

## Discussion

Falls among older people remain a major public health problem world-wide. Substantial inroads are being made, with growing research evidence of single, multiple and multifactorial interventions being effective in reducing falls. However, the results of this focussed review of the subgroup of falls prevention randomized trials for the community setting conducted in countries in Asia, where 60% of the world’s population live, indicates substantial gaps. Not only has there been limited falls prevention research conducted in Asian countries, but where studies have been conducted, sample sizes are generally small, and only 11 of the 30 interventions evaluated in the 26 studies in Asia achieved a significant reduction in one or more falls outcomes (37%). The meta-analysis results where more than one study was able to be pooled for an intervention type (exercise and multifactorial interventions) indicated that only exercise was effective in reducing falls in Asian populations.

Exercise is by far the most researched intervention type in Asian countries (as it is world-wide), with over half of the studies reported having an exercise component. Not surprisingly, Tai Chi was commonly investigated, and was effective in reducing falls outcomes in three studies in Asian countries, and in the sub-group meta-analysis. Although Tai Chi has been shown to be acceptable [56] and to reduce falls-related outcomes in non-Asian countries, [3] it does seem to be a preferred exercise approach for investigation and implementation in Asian countries. Adherence to the successful Tai Chi programs conducted in Asia was high (75–81%), though similarly high adherence to Tai Chi has been reported in studies conducted in other countries (68–80%) [57, 58]. Of note though, nine of the 15 studies investigating exercise in Asian countries were not effective, and the majority of these did not meet the criteria recommended by Sherrington [54] to be likely to be effective in reducing falls (having a moderate challenge to balance, and minimum of 50 h exercise dosage). Three studies that did meet these criteria were not effective, but were substantially underpowered to identify a reduction in falls related outcomes [29, 47, 52]. Other exercise approaches that were effective in single studies were an obstacle course, [43] a multi-target stepping program, [44] and a group balance and strength training program [46].

In an updated meta-analysis by Sherrington published around the time of this paper’s publication, more stringent dosage criteria (greater than three hours per week) were identified as achieving the greatest benefit in reducing falls (23% reduction) [59]. Higher exercise dosages, and sustained exercise (lifelong behaviour change) are clearly more desirable. However, low levels of sustained participation have been reported in falls prevention exercise programs, [60] suggesting that strategies such as starting off with lower dosages and gradually building up, and embedding behaviour change elements into exercise programs to support sustained and increased participation may be required to achieve this high dosage. Future exercise studies should aim to adopt a method that meets the updated Sherrington criteria while being culturally relevant, as well as being adequately powered for falls outcomes.

These results have substantial implications for falls prevention research and practice in Asia. It is often assumed that interventions shown to be effective in randomized trials or meta-analyses in one country will be generalizable elsewhere. However, researchers in the falls prevention area, [23, 61] and in other areas of health (eg hepatocellular carcinoma) [62] have called for local research in Asian and or developing countries to address key gaps and differences. In this context, recent research has also highlighted the diverse range of factors that may complicate or reduce likely effectiveness of directly translating falls prevention approaches found to be effective in non-Asian countries into Asian countries [18, 19, 23]. Some of these differing factors include: (1) role of family (including filial piety); (2) indoor (including floor surfaces) and outdoor environments; (3) regularly worn footwear in many parts of Asia differ from what is considered the ideal footwear for falls prevention; (4) lifestyle factors such as incidental and formal exercise approaches that are routine and acceptable, diet, and sunlight exposure; (5) health services and systems, and patient expectations of specific health practitioners; (6) differing understanding of prevention and active engagement in prevention and intervention approaches; and (7) differences in concern about falls influencing behaviours [23, 61, 63, 64].

Given the factors outlined above, direct translation of interventions from non-Asian studies to Asian countries may warrant careful consideration. There are a number of implications for researchers, practitioners, policy and planning personnel, and research funders who may be involved in future falls prevention research in Asia. Firstly, in the area where the meta-analysis of Asian studies indicated effective interventions (Tai Chi and other exercise approaches), and where there is at least one effective randomized trial (home modifications, multiple and multifactorial interventions), that these interventions could be considered for broader implementation into practice. Two other interventions were shown to be effective in special populations: vitamin K2, vitamin D2 and calcium supplementation for women with probable Alzheimer’s disease; and a multifactorial intervention for post hip fracture surgery patients. Even in these areas where there is some evidence of effectiveness, there is a need for research with larger samples (as the majority in this review were small samples, which limits the rigor and confidence in study findings). Furthermore, in areas like exercise, the different exercise intervention types (other than Tai Chi) have been grouped together in this review (because of the small number of studies), so there remains scope for further research exploring other exercise modalities, particularly those that may be most acceptable to Asian populations.

Secondly, for other areas of practice where there is research evidence of effective interventions in non-Asian populations but not in Asian populations, a number of options are available. For those interventions where research has been conducted in Asian countries but was not shown to be effective (eg knowledge/education interventions, and psychological interventions) there is a clear need to review these unsuccessful methodologies in the context of local factors that may influence their uptake and effectiveness. For example, critical elements to a successful falls prevention education program include that participants understand that falls are preventable, and that changes in behaviour, even at later ages, can still improve risk of future falls. However, research indicates that in some Asian cultures (eg China), fatalistic beliefs about falls is a major barrier that would need to be overcome [22]. Researchers and practitioners need to undertake research to improve understanding of these beliefs, and strategies that may influence these beliefs. Recent research in Australia has shown a World Café approach to be valuable in informing understanding of factors that would influence uptake and sustained engagement in falls prevention among older people, [65] and these factors have been introduced into a peer education program that was effective in increasing intention to engage in falls prevention activities [66]. Innovative, culturally relevant approaches to understanding these factors in Asian countries, and approaches to achieve sustained behaviour change (which may include these or other culturally relevant approaches) are foundational to achieving improved falls related outcomes. In using results from these type of local studies, practitioners can implement these interventions in a blended manner that retains as much of the original intervention approach as possible, but with local tailoring. For other types of falls prevention interventions that have not been investigated in Asian samples (eg medication review/reduction) there would be merit in establishing local factors as described above, prior to researchers in Asia seeking funding for local research to evaluate a culturally tailored intervention’s effectiveness in reducing falls or falls injuries. In the context of medication review, inclusion of traditional and herbal medicines (which are widely used in some Asian countries), as well as interactions with pharmacy medicines would be important considerations. There may be value for this research to be collaborative with researchers who have implemented effective randomized controlled trials in non-Asian countries.

Finally, there may be other novel, locally relevant intervention types that have not been researched, that may warrant funding being sought for these interventions to be evaluated in an Asian context.

For the purposes of this research we have grouped countries under the broad umbrella classification of “Asia”, however it is important to recognize that there is considerable diversity between some of the countries in Asia, and even within some countries (for example, in Malaysia where there are three significant ethnic populations), or between urban and rural populations, where socioeconomic and other differences in some Asian countries can be stark. Specific understanding of local, cultural and societal factors that may influence acceptability of interventions need to be considered when implementing falls prevention interventions in Asian countries.

Although we have identified only a relatively small number of randomized controlled trials investigating falls prevention approaches in Asian countries, there appears to be a steady growth in the number, size and quality of the studies published more recently (since the 2012 Cochrane review) [3]. There are also two protocol papers published for studies that are underway in Asia, including a multifactorial intervention for older people with recent history of falls or injuries from falls in Malaysia (*n* = 300), [67] and an evaluation of the use of exercise using Nintendo® Wii in Singapore (*n* = 80), [68] that will add to the small but growing volume of falls prevention research in this region. Of note, a number of the more recent studies are substantially larger than the median sample size of 150 for studies from Asian countries reported in the Cochrane review, which strengthens confidence in the study findings. However, the majority focussed specifically on exercise interventions only, and all were from more well developed countries in Asia.

There were several limitations to this review. A moderate limitation given the focus of this review on studies conducted in Asian countries is that only studies published in English have been included, therefore some Asian studies published in languages other than English may not have been identified. Another limitation is the varying falls outcomes published, which did not allow for additional meta-analyses to be undertaken. It would be beneficial for future falls prevention studies to include standardized outcomes to make direct comparisons and meta-analyses possible.

## Conclusions

In summary, this focussed review of community setting falls prevention randomized controlled trials conducted in Asia found limited evidence of a small number of effective intervention types, relative to the strong evidence across a range of intervention types from non-Asian countries. Exercise approaches have had the strongest level of investigation, and several exercise approaches, in particular Tai Chi, have been shown to be effective. There is a need for substantial investment in large, adequately powered randomized controlled trials evaluating falls prevention interventions across Asia, in particular that incorporate tailoring of intervention approaches to the local Asian context, in order to reduce the projected escalating impact of falls in this rapidly aging part of the world.

## Additional file

Additional file 1: PRISMA checklist. (DOC 64 kb)

## Abbreviations

CBT: Cognitive Behavioral Therapy
OR: Odds Ratio
ProFaNE: Prevention of Falls Network Europe
RCT: randomised controlled trial
